# Supplementary material for: Development of a computer-aided design software for the quantitative evaluation of aesthetic damage
Source: PLoS One. 2019 Dec 18;14(12):e0226322. doi: 10.1371/journal.pone.0226322 (PMC6919621; doi:10.1371/journal.pone.0226322)
Supplement: S1 Code site — 1. (ZIP) [file pone.0226322.s001.zip › [EESC_jr][Projeto]Relatorio_07_21/reports/templates_zip/gentelella-master/vendors/autosize/example/index.html]

Simple Autosize for textareas


### max-height 300px

The coconut palm (also, cocoanut), Cocos nucifera, is a member of the family Arecaceae (palm family). It is the only accepted species in the genus Cocos.[2] The term coconut can refer to the entire coconut palm, the seed, or the fruit, which, botanically, is a drupe, not a nut. The spelling cocoanut is an archaic form of the word.[3] The term is derived from 16th-century Portuguese and Spanish coco, meaning "head" or "skull",[4] from the three small holes on the coconut shell that resemble human facial features.

### no max-height

The coconut palm (also, cocoanut), Cocos nucifera, is a member of the family Arecaceae (palm family). It is the only accepted species in the genus Cocos.[2] The term coconut can refer to the entire coconut palm, the seed, or the fruit, which, botanically, is a drupe, not a nut. The spelling cocoanut is an archaic form of the word.[3] The term is derived from 16th-century Portuguese and Spanish coco, meaning "head" or "skull",[4] from the three small holes on the coconut shell that resemble human facial features.
